# Dietary Calcium and Vascular Calcification: The Way to One’s Heart Isn’t Always Through the Stomach

**DOI:** 10.1016/j.jacadv.2024.100994

**Published:** 2024-05-21

**Authors:** Jared Alexander Spitz, Lily Nedda Dastmalchi

**Affiliations:** a Inova Schar Heart and Vascular, Fairfax, Virginia, USA; b Department of Medicine, Heart and Vascular Institute, Temple University Hospital, Philadelphia, Pennsylvania, USA

**Keywords:** Atherosclerosis Risk In Communities study (ARIC), coronary artery calcification (CAC), dietary micronutrients, extra-coronary calcification (ECC)

**Figure:**
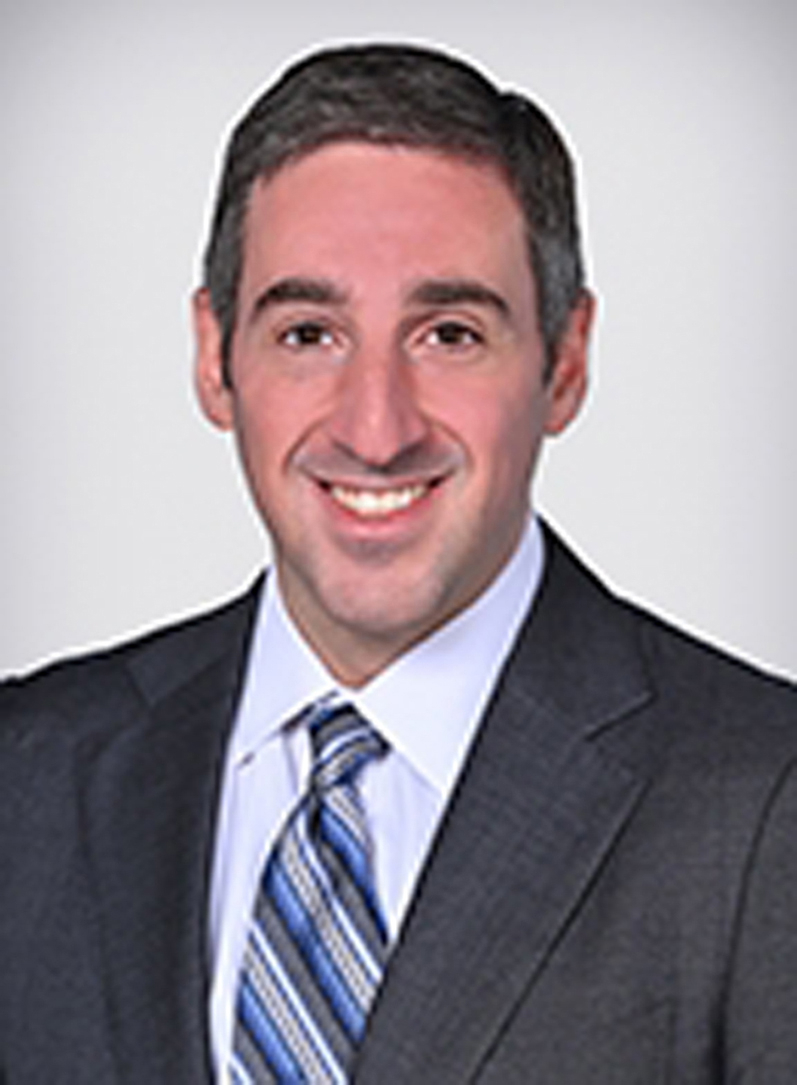

Calcium and phosphorus are essential for bone and overall health.^1^^,^^2^ Individuals need to consume adequate amounts with daily requirements varying by age and gender.^1^^,^^3^ Counterbalancing this is evidence of adverse cardiovascular disease (CVD) outcomes in those with higher levels of serum calcium and phosphorus.^2^^,^^4^ There is concern that excess dietary intake may mediate these adverse events by promoting vascular calcification with the attendant consequences of atherosclerotic CVD.^5^ Cohort studies like the Framingham Offspring Study^6^ and the MESA (Multi-Ethnic Study of Atherosclerosis)^5^ have illustrated that dietary calcium intake may not be a contributor to coronary artery calcification (CAC)^5^ or increased incidence of CV events.^6^^,^^7^ In fact, 1 cohort study suggests that increased risk of CVD and myocardial infarction from excess calcium is driven by supplemental calcium intake.^4^ Given the discrepant findings from these studies, there is a need to clarify the relationship between dietary calcium and phosphorus intake and incident CAC.

In this issue of *JACC: Advances*, Nohara-Shitama et al^8^ present a new approach to assessing the relationship between dietary calcium and phosphorus intake with incident CAC and extra-coronary vascular and valvular calcification (extra-coronary calcification [ECC]). Using participants from the previously described ARIC (Atherosclerosis Risk In Communities) study,^9^ 1,914 individuals without known coronary heart disease (CHD) were chosen who had undergone serial dietary assessment in middle age at their initial visit and 10 years later during their third visit using a validated food frequency questionnaire (FFQ). They subsequently had computed tomography scanning done approximately 20 years later as part of the study. The primary outcome was the association of dietary calcium and phosphorus with CAC and ECC, which included the ascending aorta, descending aorta, aortic valve ring, aortic valve, and mitral valve.

The investigators found an inverse relationship between the amount of dietary calcium with CAC and ECC, specifically the aorta and aortic valve ring. Participants with the highest consumption of dietary calcium were found to have a statistically significant lower amount of calcification of the 3 vascular beds, further supporting the notion that risk factors for atherosclerosis can vary by vascular location.^10^^,^^11^ Dietary phosphorus intake showed a similar inverse relationship with CAC; however, there were no significant findings with ECC (Figure 1). Interestingly, there was a stronger association seen in men vs women (for example, increased phosphorus was inversely associated with lower aortic valve ring calcification in men vs women) although no statistically significant interaction was seen between the genders. The study supports the impact gender has on the development of CVD.^12^

**Figure 1:**
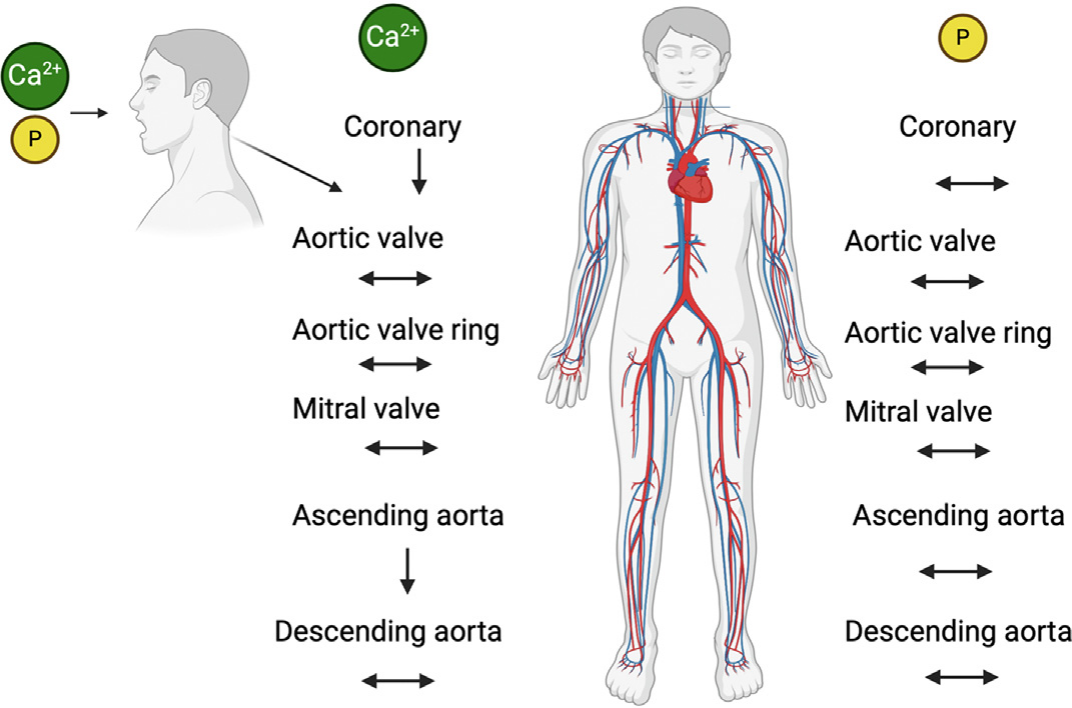
**Dietary Calcium and Phosphorus Intake with Vascular and Valvular Calcification** The association of the highest quartile of dietary calcium and phosphorus intake with coronary and extra coronary calcification. The highest quartile of dietary calcium Is inversely associated with coronary, aortic ring, and ascending aortic calcification; the highest quartile of phosphorus intake Is inversely associated with coronary artery calcification. Downward arrow = statistically significant inverse association of highest and lowest intake quartiles; horizontal arrow = no statistically significant difference between highest and lowest quartiles; Ca^2+^ = calcium; P = phosphorus.

There are several strengths to this study that are notable. The majority of the studied population was female; therefore, we believe the results can apply to both genders. Previous research has relied on dietary information from a single point in time. This can allow for significant misestimation of dietary intake. The authors note that the highest median reliability coefficient for the FFQ was 0.70 for phosphorus intake in White men and as low as 0.26 for calcium intake in Black women. The authors leverage the longitudinal dietary data from ARIC to average intake over 2 separate time points to account for this potential bias and strengthen the reliability of the dietary assessment. Another strength of the study is the evaluation of ECC. While certain risk factors are common to atherosclerosis across different vascular beds (ie, hyperlipidemia, hypertension, smoking), the relative impact on the development of atherosclerosis differs by risk factor in any given vascular bed.^10^^,^^11^ This paper begins to ask the question about the differential effects of dietary calcium and phosphorus in coronary and noncoronary beds. Additionally, valvular calcification appears to be partially mediated by risk factors distinct from those associated with vascular calcification.^13^

Several weaknesses warrant mention. While micronutrient consumption is imperative in growth and development, dietary patterns across the lifespan have been shown to be most associated with CVD and outcomes.^14^ High dietary sources of calcium and phosphorus are found in animal products and dairy.^1^^,^^15^ Interestingly, the diets most supported by the American College of Cardiology/American Heart Association recommend avoiding red and processed meats, low amounts of fish and poultry, and minimal consumption of low fat dairy products.^15^ Using the FFQ in 2 points in time prior to imaging may not be reflective of someone's true dietary pattern over the life course. Red meat, dairy, and vegetable consumption may have impacted dietary phosphorus and calcium intake, which may impact outcomes if not controlled for in the present analysis.

Additionally, these patients were without baseline CHD and may represent an overall healthier population. Perhaps the biggest limitation is the lack of serial CAC or ECC evaluation. The authors evaluate changes in dietary intake on CAC and ECC but do not assess change over time. Notably, a study in MESA of 5,448 individuals without CHD evaluated the relationship between dietary (and supplemental calcium intake) with incident CAC over time with CAC testing performed ∼10 years later in about half of the patient cohort. In their analysis, elevated total calcium intake was associated with a decreased incidence of CAC; in patients with CAC >0 at baseline, there was no association between calcium intake and worsening CAC.^5^ The last notable limitation is that the current study does not have cardiovascular outcome data. Therefore, this analysis is limited to surrogate endpoints of vascular calcification.

The authors should be congratulated on their contribution to this question. Frequently, clinicians are faced with the question about dietary calcium as a contributor to CAC score. There are now a number of well-done studies that evaluate this question. The researchers’ findings suggest that dietary calcium and phosphorus intake may have an inverse relationship to the development of CAC and ECC. However, further studies with more precise dietary information, attention to gender, and attention to different vascular beds are needed to clarify these relationships.

## Funding support and author disclosures

Dr Dastmalchi has served on the advisory board for Novartis. Dr Spitz has reported that he has no relationships relevant to the contents of this paper to disclose.
